# Modeling the relationship between digital nativity and Smartphone usage in learning English as a foreign language contexts

**DOI:** 10.3389/fpsyg.2022.1053339

**Published:** 2023-01-11

**Authors:** Lianghong Hui, Lin Sophie Teng, Fangfang Guo

**Affiliations:** ^1^Department of English, Northeastern University, Shenyang, China; ^2^Department of Foreign Languages and Literatures, Tsinghua University, Beijing, China; ^3^Department of Linguistics, Zhejiang University, Hangzhou, China; ^4^Department of College English Teaching, Hebei Normal University of Science and Technology, Qinhuangdao, China

**Keywords:** digital nativity, Smartphone usage, structural equation model, English learning, tertiary education

## Abstract

**Introduction:**

Despite many studies exploring the application of digital devices in foreign language learning, only some have investigated the influencing mechanisms of digital nativity on Smartphone usage in this increasingly seamless learning environment. This research aims to explore the relationships between college students’ digital nativity and their use of Smartphones for English learning.

**Methods:**

The data were collected from 502 undergraduates in mainland China through self-reported questionnaires, namely the Digital Natives Assessment Scale and the Smartphone Use in Learning Foreign Language Scale.

**Results and Discussion:**

The confirmatory factor analysis validated a four-factor measurement model of digital nativity, including “grow up with technology”, “comfortable with multitasking”, “reliant on graphics for communication” and “thrive on instant gratification and rewards”. A second-order measurement construct of favorable Smartphone usage and its first-order adverse effects in foreign language learning were also examined, demonstrating good validity and reliability. Structural equation modeling analysis revealed that students who displayed more attributes of “grow up with technology” and “thrive on instant gratifications and rewards” tended to adopt smartphones positively for English learning. In addition, those who were more familiar with technological assistance might suffer less from the adverse effects of Smartphone usage. However, the preference for immediate responses and feedback could also lead to more adverse effects when using Smartphones for English learning. Besides, “comfortable with multitasking” and “reliant on graphics for communication” didn’t have any significant predictive impact on either the favorable functions or the adverse effects of Smartphone usage. Based on the research results, we discuss the theoretical and practical implications.

## 1. Introduction

In the past two decades, the characteristics of digital natives have aroused broad interest in educational academia, probing either the attributes (Prensky, 2001; Helsper and Eynon, 2010; Teo, 2013) or the influencing mechanisms (Chen et al., 2016; Çoklar et al., 2017; Yurdakul, 2018; Wang et al., 2019; Calvo-Ferrer, 2020; Fiedler et al., 2022) of this burgeoning term. A growing body of evidence has accumulated showing the importance of digital nativity for college students learning (Lei, 2009; Tapscott, 2009; Bennett and Maton, 2010; Margaryan et al., 2011). Prensky (2001) suggests that the brain structures of digital natives are different from their predecessors because of their neuroplasticity nature shaped by the frequent interaction with technologies. Bennett and Corrin (2019) noted that digital natives think, behave, and learn differently from digital immigrants. There is no doubt that digital natives demonstrate a tremendous disparity from the digital immigrants in learning styles (Bennett et al., 2008), habits (Zimerman, 2012), and preferences (Helsper and Eynon, 2010) in the technology-rich environment of modern learning. Despite the popularization of this concept, there is a lack of research examining the influence of digital nativity on using Smartphones for English learning, which has been increasingly reliant on the advancement of digital technology.

Growing up in the mobile world, Smartphone has become an indispensable necessity for today’s digital natives in their daily communication, entertaining, and learning. Hence, during the past years, foreign language teachers and educators have witnessed the widespread tendency of Smartphone usage to assist language teaching around the world (Lu et al., 2016; Botero et al., 2018; Wan et al., 2018; Andujar, 2020; Chen et al., 2020). Smartphone is deemed a practical and suitable digital device to facilitate foreign language proficiency in this increasing knowledge explosion era. On the one hand, this benefit is highly attributed to the affordability and accessibility of Smartphone and internet connections (Şad et al., 2020; Wrigglesworth, 2020). On the other hand, there is an agreement among language teaching practitioners and researchers that the audiovisual presentation of language learning materials is proven to be more engaging and interactive in helping language learners immersed in an authentic learning environment seamlessly (Kim and Kwon, 2012; Andujar, 2016; Lekawael, 2017; Hwang and Fu, 2018). Just as pointed out by Prensky (2005) in his observation about the ubiquitous power of the cell phone that “cell phones complement the short-burst, casual, multitasking style of today’s digital natives.” However, there is a lack of empirical investigation on the relationship between digital nativity and Smartphone usage in college students’ foreign language learning, even though there exists significant literature in support of digital nativity as an antecedent for online behaviors and proficiency among college students (Chen et al., 2016; Çoklar et al., 2017; Yurdakul, 2018; Wang et al., 2019; Calvo-Ferrer, 2020).

Given the research gaps mentioned above, this study explores how different digital attributes predict both beneficial and detrimental effects of Smartphone usage. Structural equation modeling analysis was used to examine the relationships of digital nativity with favorable Smartphone usage and its adverse impact separately. We postulate that the granular exploration into the predicting effects of digital nativity would enhance our insights into the influencing mechanisms of digital native features as significant antecedents in affecting students’ use of Smartphones to learn English.

## 2. Literature review

### 2.1. Digital nativity and its effect on learning

Digital nativity is the multi-dimensional construct of psychological characteristics and behavioral tendencies possessed by those fluent in technological practices. Teo (2013) conducted a pioneering empirical study to develop a psychometric scale for the measurement of digital nativity with four attributes, namely “grow up with technology,” “comfortable with multitasking,” “reliant on graphics for communication,” “thrive on instant gratifications and rewards.” This scale was further validated in different contexts cross-culturally (Chen et al., 2016; Çoklar et al., 2017; Yurdakul, 2018; Wang et al., 2019; Calvo-Ferrer, 2020) with different ages (Huang et al., 2021). Furthermore, some findings revealed that age does not distinguish digital natives from digital immigrants (Helsper and Eynon, 2010; Zhao and Zhao, 2021).

Previous studies have generally established the relationship between digital nativity and online learning tendencies and behaviors (Chen et al., 2016; Çoklar et al., 2017; Yurdakul, 2018; Wang et al., 2019; Calvo-Ferrer, 2020; Zhao and Zhao, 2021); however, the relational directions among these variables are unclear. To be specific, digital nativity was found to be correlated positively with adaptive online behaviors, such as, online information search strategies and information literacy (Çoklar et al., 2017; Aharony and Gazit, 2019), pre-service teachers’ technological pedagogical content knowledge (TPACK; Yurdakul, 2018), teachers’ technology adoption (Zhao and Zhao, 2021), and sustainability education outcomes (Fiedler et al., 2022). On the contrary, digital nativity has also been found to be related to maladaptive behaviors, such as distraction of academic motivations (Chen et al., 2016) and technological additions (Wang et al., 2019). Therefore, whether digital nativity has positive or negative effects on people’s online behaviors is still blurred. To our knowledge, only two studies have explored the effect of digital nativity on students’ English learning but yielded inconsistent results (Calvo-Ferrer, 2020; Wang et al., 2022). Calvo-Ferrer (2020) finds out that in an online gamified learning context, the attribute of “multitasking” negatively predicts students’ English vocabulary learning, while “grow up with technology” and “thrive on instant gratifications and rewards” are positively related with students’ vocabulary learning outcomes. But “reliant on graphics for communication” is not correlated with their vocabulary learning achievement. Wang et al. (2022) reported that digital nativity was positively related to students’ online self-regulation and English learning engagement. Therefore, despite the importance of digital nativity in understanding students’ learning patterns and behaviors in technology-rich environments (Tapscott, 1999; Prensky, 2001; Teo, 2013; Tran et al., 2020; Huang et al., 2021; Fiedler et al., 2022), there is still a shortage of studies examining the effects of digital native attributes on students’ English learning practices and attitudes in digital environments (Calvo-Ferrer, 2020).

### 2.2. Smartphone usage in foreign language learning

Smartphones have many advantages for university students’ foreign language learning. On the one hand, the portability and multi-functions of Smartphones have dramatically contributed to their dominance in mobile-assisted language learning (MALL). Besides, communicative function based on advanced technology in automated corrective feedback generation and augmented reality enhanced the interactive efficiency (Wrigglesworth, 2020). The multimodal display (Pachler et al., 2010) and the gamification features can easily arouse the enthusiasm of application users, stimulating their innate motivation for language learning (Calvo-Ferrer, 2015) and turning the monotonous drills of language structure exercises into an engaging and appealing experience (Purgina et al., 2020).

Despite the prevalence of Smartphone usage, with some studies revealing the positive effects on foreign langue learning, there exist a discrepancy between learners’ perceptions about and the actual use of Smartphone apps in their English learning (Şad et al., 2020; Metruk, 2022). For example, potential learning distraction (Metruk, 2020), unethical cheating in exams (Ugur and Koç, 2015), and insufficient readiness for technology adoption among teachers (Dashtestani, 2013) are the three significant drawbacks of incorporating mobile technology into classrooms (Metruk, 2020). Other obvious problems are the physical features of Smartphones in terms of the small screen size (Dashtestani, 2013; Arinder, 2020), unstable internet access, and financial and technological challenges (Burston, 2014; Ekinci and Ekinci, 2017) as well as the lack of guidance for students using Smartphones productively (Abdullah et al., 2019).

To comprehensively examine first-year students’ views toward Smartphone usage in English learning, Şad et al. (2020) designed a scale to investigate both the pros and cons of Smartphones in modern youth lives concerning their foreign language learning. The scale consists of four dimensions, inspecting both the positive and negative influence of Smartphones on college students’ English learning. The four dimensions are “General Contribution,” “Reading and Writing,” “Speaking and Listening” and “negative effects” This instrument provides a comprehensive tool to inspect college students’ Smartphone practices in their English learning.

Based on the above analysis, we argue that the first three dimensions of Smartphone usage may converge on a higher-order construct, namely favorable Smartphone usage. The last dimension of adverse effects may form a distinct factor. In addition, the positive and negative effects of Smartphones are conceptually different constructs, which formulate innately contradictory relationships (Şad et al., 2020; Metruk, 2022). The proposed second-order factor has some merits, such as parsimony, the avoidance of bandwidth-fidelity dilemma, and collinearity reduction (Sarstedt et al., 2019). Therefore, it is sensible to distinguish the favorable effects from the adverse effects of Smartphone usage while examining their predictive power on digital nativity.

### 2.3. The relationship between digital nativity and Smartphone use

Some researchers (e.g., Hwang and Fu, 2018) have argued that more attention should be given to the affective or psychological states of learners in mobile-assisted language learning processes. Digital nativity is a psychometric attribute with different levels among individuals than a generational trait (Teo, 2013). Hence, it is illuminating to explore the relationship between the psychometric properties of digital nativity and students’ Smartphone usage in English learning.

First, Teo (2016) summarized that digital natives were more efficacious in using state-of-the-art technologies. Thongsri et al. (2020) discovered that Chinese students’ computer self-efficacy positively affected college students’ scores and satisfaction in using mobile phone applications to improve their English vocabulary. Their findings indicate that those confident in their computer capability are more likely to explore the advanced functions of mobile devices. In addition, Şad et al. (2020) discovered that the more time college students spent online, the more frequently they displayed using Smartphones to do listening and speaking activities and suffered more adverse effects from Smartphone usage. Regarding second language vocabulary acquisition, “grow up with technology” positively predicated both short-term and long-term gains (Calvo-Ferrer, 2020). Therefore, it is reasonable to presume that the attribute of “grow up with technology” positively influences “favorable Smartphone usage” as well as “adverse effects.”

Second, previous studies show that the relationship between multitasking and learning is still blurred. For example, research proved that multitasking negatively influences online learning (Burak, 2012). In addition, multitasking is a significant distraction for learning with mobile phones (Chen and Yan, 2016; Klimova, 2019). In other investigations, multitasking is negatively associated with learning achievement (Junco and Cotton, 2012; Ravizza et al., 2014). Specifically, multitasking negatively predicted students’ English vocabulary acquisition in both short-term and long-term retention (Calvo-Ferrer, 2020). But, in one empirical research, there was no relationship between students’ rate of correct answers after watching video material and using mobile phones for texting during the watching process (Lawson, 2013). In addition, “comfortable with multitasking” cannot predict Smartphone addiction significantly (Wang et al., 2019). Because of the mixed results of previous findings, more empirical evidence is needed to explore the specific effects of multitasking on learning. Then, we propose that “comfortable with multitasking” negatively influences “favorable Smartphone usage.” But “comfortable with multitasking” positively influences the “adverse effects.”

Prensky (2001) believes that “reliant on graphics” is an adaptive feature for digital natives because of the neuroplasticity in their brains. What’s more, Lowe and Pramono (2006) found that pictures and annotations are facilitating factors for understanding complex and dynamic information. Besides, other empirical evidence reveals that high graphic and video annotation usage leads to significant high achievement and retention in foreign language vocabulary learning, indicating the effectiveness of graphics and videos in vocabulary acquisition (Gürkan, 2018). Nevertheless, “reliant on graphics” is reported to positively predict all four types of information technology addiction (Wang et al., 2019). Consequently, we postulate that the effects of “reliant on graphics for communication” are very complex. “Reliant on graphics for communication” may positively affect “favorable Smartphone usage.” It may also positively influence “adverse effects” in learning English.

Previous survey results reveal that “thrive on instant gratifications and rewards” is associated significantly with Internet gaming disorder and Smartphone addiction, indicating this dimension’s negative effect on learning. Additionally, Chen et al. (2016) presumed that thriving on instant gratification and rewards would interfere with students’ academic learning motivation, which negatively affects students’ school decisions. According to Bembenutty and Karabenick (2013), delay of gratification, as the opposite concept of instant gratification, is positively associated with students’ self-regulation, which is a significant predictor of students’ academic achievements (Zheng and Li, 2016). Despite the adverse effects, “thrive on instant gratifications and rewards” was discovered to influence students’ short-term English vocabulary acquisition, while having no predictive impact on the long-term gains (Calvo-Ferrer, 2020). Therefore, we hypothesize that “thrive on instant gratifications” negatively influences “favorable Smartphone usage.” However, “thrive on instant gratifications” positively affects “adverse effects” in learning English.

### 2.4. The present study and research questions

With the prominent role of digital nativity in shaping young adults’ daily behaviors increasingly recognized in the literature (Prensky, 2001; Teo, 2013; Çoklar et al., 2017; Wang et al., 2019), the relevant exploration can be further extended in more aspects. First, there is scarce research exploring effects of digital nativity on students’ learning behaviors in the technological environment. It is also essential to examine the separate impact of digital nativity on students’ use of technology (Prensky, 2001). That’s because most previous studies have explored digital nativity from a holistic perspective (Çoklar et al., 2017; Yurdakul, 2018; Fiedler et al., 2022), which can not reveal the precise influencing mechanisms of individual digital characteristics. Therefore, it is important to take each factor as distinct psychological characteristics or behavioral tendencies (Wang et al., 2019).

On the other hand, the influence of different attributes may not display the same influencing pattern. Thus, it is necessary to investigate the effects of individual digital native characteristics so that a clear directional influence of each predictor can be manifested. Second, although the use of Smartphones in foreign language learning has aroused increasing attention among scholars (Nami, 2020; Zou et al., 2022), there has been comparatively little attention to the use of Smartphones from the perspective of digital nativity as antecedents. Third, unlike the Digital Natives Assessment Scale, which has been validated by many studies in China (Teo, 2016; Huang et al., 2021; Zhao and Zhao, 2021) and western countries (Çoklar et al., 2017; Yurdakul, 2018; Calvo-Ferrer, 2020; Fiedler et al., 2022), the “Smartphone Use in Learning Foreign Language Scale” has primarily been designed in Turkish environment. Consequently, the validity and reliability of this scale need to be examined with more empirical data in a broader cultural context.

Based on the literature mentioned above, it is critical to specify the multidimensions of both digital natives and Smartphone use in English learning context and explore the complex associations between four attributes of digital nativity and favorable and adverse effects of Smartphone use. This study aims at answering the following research questions (RQs):

1. What is the factorial structure of digital natives for Chinese EFL learners?
2. What is the factorial structure of Smartphone usage in learning a foreign language?
3. To what extent does digital nativity predict Smartphone usage.

## 3. Materials and methods

### 3.1. Participants

A total of 502 valid responses from first-year undergraduates were collected in mainland China voluntarily. There were 42.4% males (*n* = 213) and 57.6% females (*n* = 289). The average age was 19 (*SD* = 0.89), ranging from 17 to 24. The respondents claimed to have used Smartphones for around 80 years (*SD* = 3.00), indicating their familiarity with Smartphone usage. The participants reported hometowns were of different categories in terms of locations (39.8% rural or small-town areas, 60.2% urban areas). The participants were divided into four major categories, among which 49.8% were from science, 24.7% were from Arts, 20.9% were from Business or Management, and 4.6% were from foreign languages. On average, students’ self-assessed family financial status was 3.72 (*SD* = 1.06), with 1 representing extremely poor and 7 indicating extremely rich. The average score means these respondents were, on average, from medium-ranking economy families.

### 3.2. Measures

#### 3.2.1. Chinese digital natives assessment scale

Chinese Digital Natives Assessment Scale (Teo, 2016) was used to measure the attributes of college students’ digital nativity. The questionnaire has both Chinese and English versions. This study used the Chinese version of the questionnaire consisting of 21 items, measuring four aspects of the digital characteristics of modern college students, namely “Grow up with technology” (5 items, e.g., I use the Internet every day), “Comfortable with multitasking” (6 items, e.g., I am able to surf the Internet and perform another activity comfortably), “Reliant on graphics for communication” (5 items, e.g., I use pictures more than words when I wish to explain something) and “Thrive on instant gratifications and rewards” (5 items, e.g., I expect quick access to information when I need it). All items were rated on a seven-point Likert scale, ranging from 1 (not at all true of me) to 7 (very true of me). The Cronbach’s α for each factor in Teo’s research (2016) was: 0.89, 0.92, 0.88, 0.87. The model fits of CFA were: χ2/*df* = 2.94; CFI = 0.94; TLI = 0.92; SRMR = 0.04; RMSEA = 0.07, indicating the reliability and validity of the instrument.

#### 3.2.2. Smartphone use in learning foreign language scale

This research used Smartphone Use in Learning Foreign Language Scale (Şad et al., 2020) to elicit Chinese college students’ use of Smartphones for English learning in general, thus it does no focus on any specific English skill. This questionnaire is composed of 21 items measuring both favorable English learning behaviors on the Smartphone and the negative influence of Smartphones on English learning. The seven-point Likert scale includes four factors, which are “General Contribution” (6 items, e.g., I do activities on English learning websites on my Smartphone); “Reading and Writing” (5 items, e.g., “I blog in English through my Smartphone”); “Listening and Speaking” (4 items, e.g., “I watch videos in English on my Smartphone”); and “Adverse Effects” (6 items, e.g., “I have difficulty in focusing on my English classes because of my Smartphone”). In the research of Şad et al. (2020), exploratory factor analysis was conducted, and four factors were extracted, accounting for about 55% of the total variance. The factor loading ranged from 0.52 to 0.84. The Cronbach α coefficients were 0.82, 0.81, 0.79, and 0.68. No confirmatory factor analysis was used to test the factorial structure. However, based on our examination of item meanings, the first three factors describe the active usage of Smartphones in facilitating students’ English learning. Therefore, a second-order measurement model was tested (Sarstedt et al., 2019), including the first three factors, which means we tested the favorable usage and adverse effects separately. Given the differences in respondents’ English proficiency, the questionnaire was translated into Chinese by a college English teacher familiar with this research and proved by a professor in applied linguistics (Brislin, 1970). Since the two scales were all adapted from previous literature, the content validity of the instruments was guaranteed (Straub et al., 2004).

### 3.3. Procedures and analyses

Before the formal data collection, a pilot test was conducted to examine the validity of the measurement. We asked six students in the target test group to complete the questionnaire. Based on the comments on the wording, clarity, and comprehensibility of the questionnaire, the researchers reworded some items. Thereafter the anonymous online survey was distributed to undergraduate students from eight universities in six different provinces (i.e., Hebei, Jilin, Liaoning, Shandong, Shanghai, and Guangdong) in mainland China. All the respondents were informed that the data were only used for research purposes. For most participants, it took 3 to 8 min to answer all the questions in Chinese.

The data were analyzed by using SPSS17.5 and AMOS 25.0. Confirmatory factor analysis (CFA) was adopted to examine the construct validity of the two scales (Jackson et al., 2009). Informed by the literature, we investigated a four-factor correlated structure of digital nativity. A second-order factor of favorable Smartphone usage and a first-order factor of adverse effects were also tested. Then the internal consistency (Cronbach alpha, AVE, and CR) was examined to evaluate the reliability of each factor.

Pearson product–moment correlations were conducted as the basis for further structural relationship building. Structural equation modeling (SEM) was then utilized to investigate the path relationships among different antecedents (digital nativity) and outcome variables (Smartphone usage). Fit indices such as the chi-square/*df* ratio, Comparative Fit Index (CFI), the Tucker-Lewis Index (TLI), the Root Mean Square Error of Approximation (RMSEA), and the Standardized Root Mean Square Residual (SRMR) were referred to as indicators for the hypothesized model. While the chi-square test is a popular way to test model fit, several limitations exist. For example, the chi-square statistic is sensitive to sample size, so it may reject the research model when the number of samples is large (Bentler and Bonett, 1980). In this research, the sample size is 502, which is relatively large. Therefore, χ2/df is an alternative to chi-square to test the model fit (Wheaton et al., 1977). Usually, indices larger than 0.90 for the CFI and TLI are acceptable; a value smaller than or equal to 0.05 of RMSEA and a number less than 0.08 of SRMR represented acceptable fit indices (Hu and Bentler, 1999; Schumacker and Lomax, 2010; Byrne, 2011).

## 4. Results

### 4.1. Validating the factorial structure of digital natives

**Figure 1:**
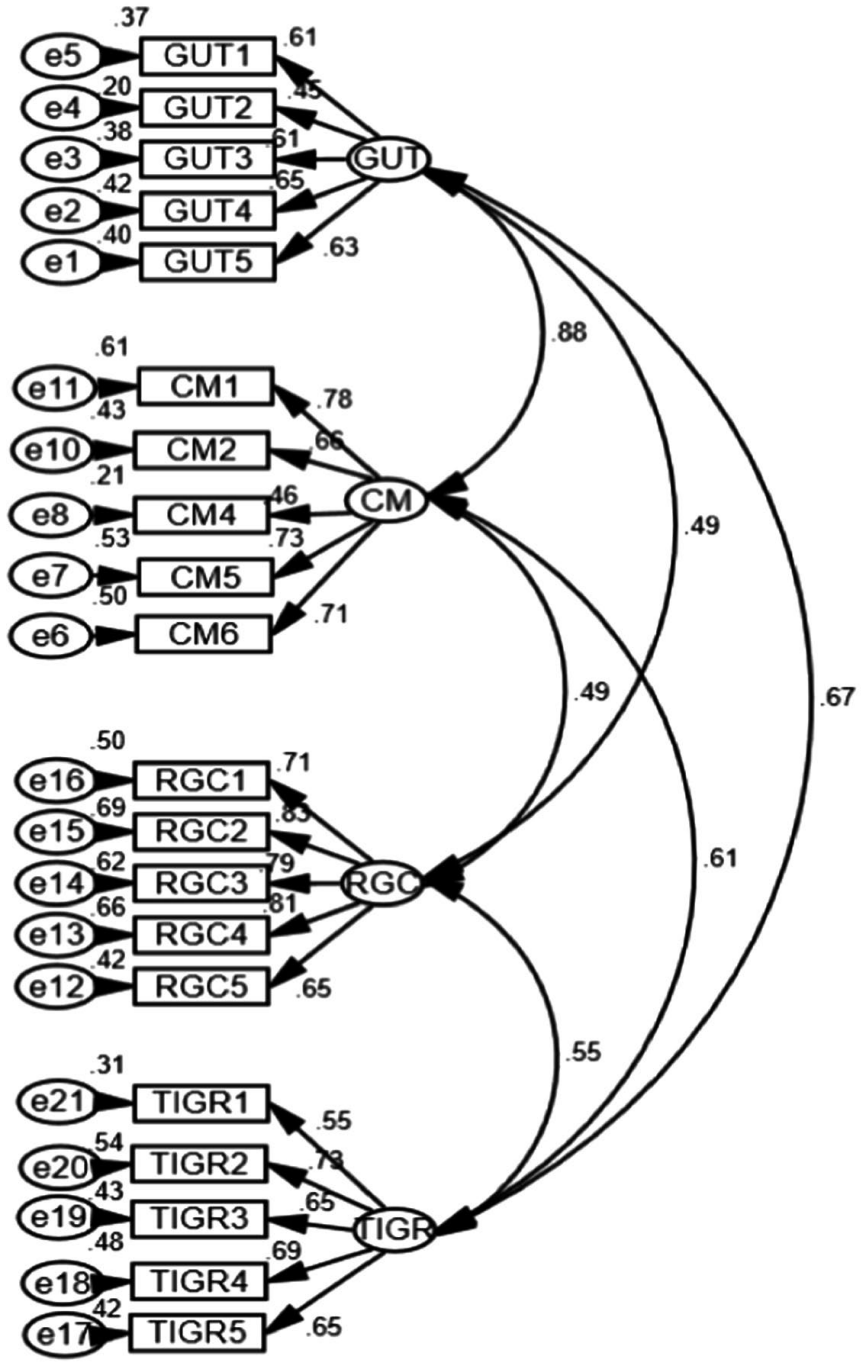
The confirmatory factor analysis result of C-DNAS (GUT, grow up with technology; CM, comfortable with multitasking; RGC, reliant on graphics for communication; TIGR, thrive on instant gratification and rewards).

We conducted multivariate normal distribution test first. The results of the skewness and kurtosis value indicated the normal distribution of the data. To test the validity and reliability of the digital nativity instrument, maximum likelihood (ML) estimation was adopted as the CFA calculation method. The measurement model fits were as follows: χ2 (183) = 602.64; χ2 /*df* = 3.29; *p* < 0.001; CFI = 0.90; TLI = 0.88; RMSEA (90% confidence interval) = 0.068 [0.062, 0.074]; SRMR = 0.060. The overall model fits values were marginally acceptable. An inspection of the standardized estimates of each item on individual factors revealed that one item loading in the “comfortable with multitasking” was below 0.45. We deleted this items which is a common practice when the factor loading of a specific item is lower than 0.5 (Comrey and Lee, 2013). Hence, this item was deleted. Subsequently, a re-run of CFA for the following 20 items generated a better model fit: χ2 (164) = 490.83; χ2/df = 2.99; *p* < 0.001; CFI = 0.92; TLI = 0.90; RMSEA (90% confidence interval) =0.063[0.057, 0.070]; SRMR = 0.056. Figure 1 presents the modified CFA measurement structure.

As shown in Table 1, the factor loading of each item is above 0.45, with the Average Variance Extracted (AVE) values between 0.35 and 0.58, which exceeds or close to the acceptable threshold of 0.36–0.50 (Fornell and Larcker, 1981). In addition, the Composite Reliability (CR) value of each factor is from 0.72 to 0.87, which is above the cut-off value of 0.70 (Nunnally, 1978; Fornell and Larcker, 1981). Besides, the Cronbach alpha values for each sub-factor ranged from 0.71 to 0.87, with the overall Cronbach alpha value being 0.90, demonstrating excellent internal consistency of the items (Table 2). Therefore, the revised measuring model of digital nativity displayed relatively good validity and reliability in this research context.

**Table 1:** The reliability of chinese digital natives assessment scale.

Factors and items	Mean	S.D.	AVE	CR	Alpha
Grow up with technology (GUT)	5.59	1.06	0.35	0.72	0.71
Comfortable with multitasking (CM)	5.10	1.36	0.56	0.80	0.80
Reliant on graphics for communication (RGC)	4.56	1.41	0.58	0.87	0.87
Thrive on instant gratifications and rewards (TIGR)	5.25	1.19	0.43	0.79	0.79

AVE, average variance extracted; CR, composite reliability.

**Table 2:** The CFA of Smartphone use in learning foreign language scale (SULFLS; *N* = 502).

Factors and items	Mean	SD	AVE	CR	Alpha
Favorable Smartphone usage	4.22	1.22	0.61	0.82	0.91
General contribution (GC)	4.86	1.41	0.51	0.86	0.88
Reading and writing (RW)	2.89	1.59	0.64	0.90	0.91
Listening and speaking (LS)	4.91	1.50	0.58	0.80	0.80
Adverse effects (AE)	3.61	1.36	0.51	0.83	0.84

AVE, average of variance extracted; CR, composite reliability; Alpha, cronbach alpha.

### 4.2. Validating the factorial structure of Smartphone use

**Figure 2:**
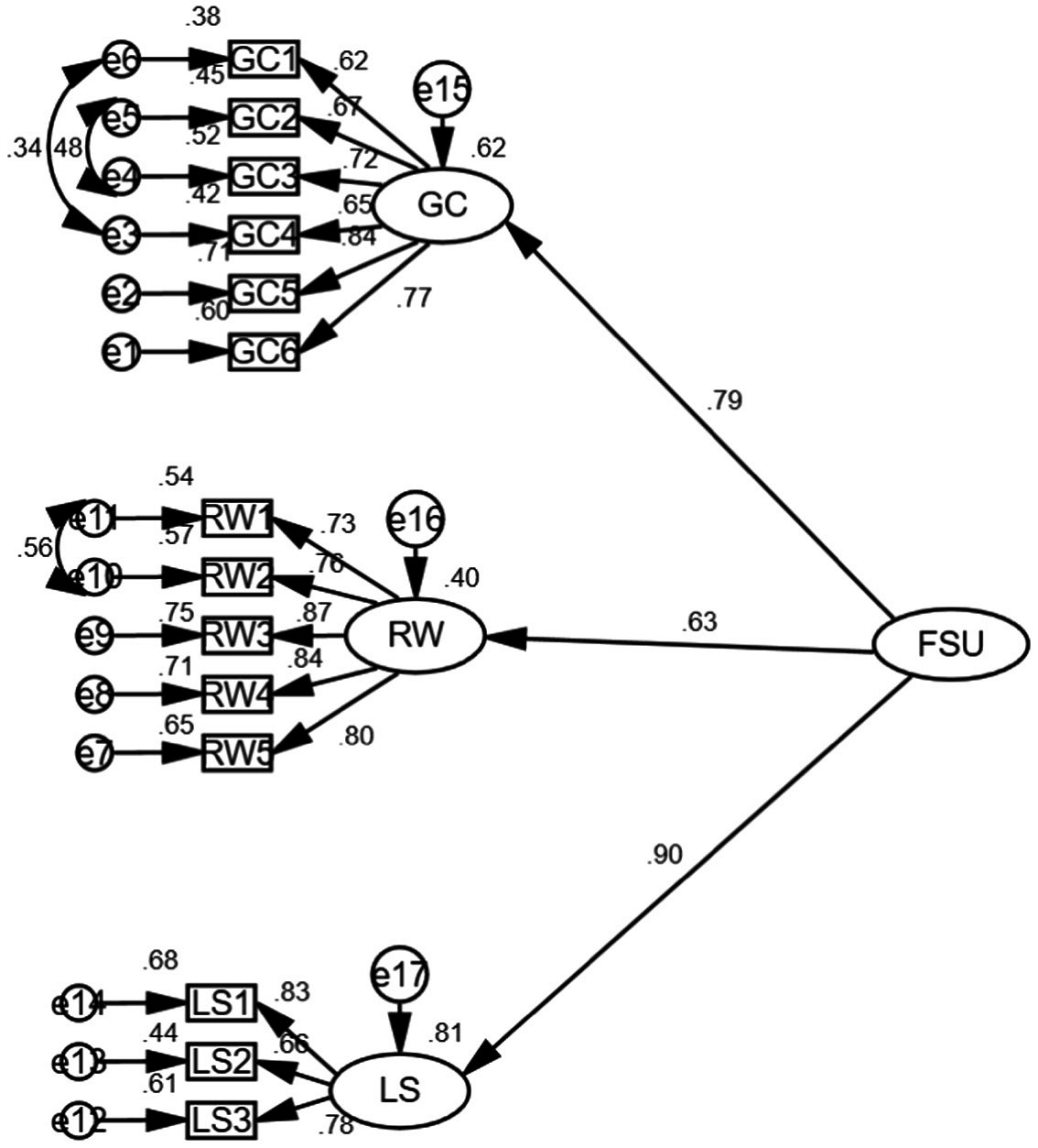
The second-order factor result of the favorable Smartphone usage scale (Note: FSU, favorable Smartphone usage; GC, general contribution; RW, reading and writing; LS, listening and speaking).

Informed by the literature review, we tested the favorable and adverse effects of Smartphone use in two different models. First, a second-order measurement construct of the favorable usage of Smartphone was examined, generating unacceptable model fit indices: χ2 (87) =914.40; χ2/df = 10.51; *p* < 0.001; CFI = 0.82; TLI = 0.79; RMSEA (90% confidence interval) =0.138 [0.130, 0.146]; SRMR = 0.10. By examining the modification indices, we noticed that the error variance of one item had a strong correlation with the error of one latent variable. This item describes learners’ practice of using Smartphones when talking with foreigners on social media. It correlates with “General contribution” which describes students’ use of apps, websites, and tutorials on their Smartphones to practice their vocabulary, listening, and pronunciation. But to make a correlation between the error of an observable variable and a latent variable violates the basic assumptions of structural equation modeling (Kline, 2016). Therefore, this item was deleted. Besides, according to the MI values, we built some correlations between the errors within the same factors. Thus, the revised model fit indices were: χ2 (71) =351.15; χ2/df = 4.95; *p* < 0.001; CFI = 0.93; TLI = 0.92; RMSEA (90% confidence interval) =0.089[0.080, 0.098]; SRMR = 0.072. These fit indices were relatively adequate. Consequently, the model was accepted, as shown in Figure 2. The factor loading of each item for favorable Smartphone usage ranged from 0.62 to 0.87, all above 0.5. The AVE values all surpassed the cut-off value of 0.5, and the CR values ranged from 0.80 to 0.90, designating an excellent convergent validity. What’s more, the total Cronbach alpha value of the scale was 0.91, with the reliability values of each sub-factor between 0.80 and 0.91, indicating good internal consistency. Therefore, the revised second-order construct of the favorable Smartphone usage scale is valid and reliable in Chinese contexts.

**Figure 3:**
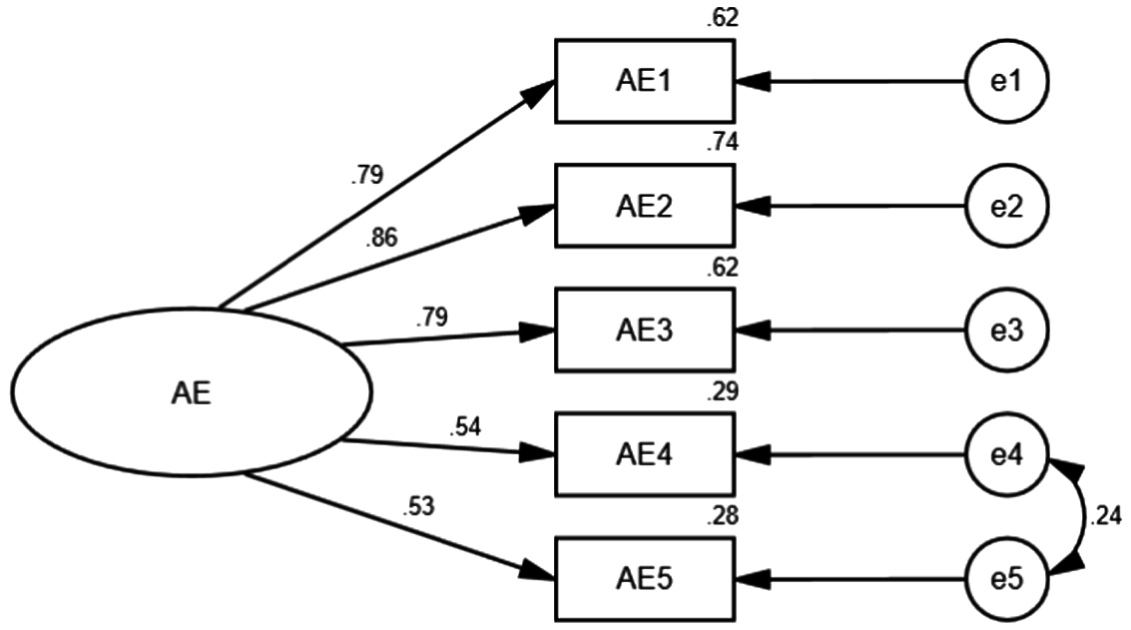
Confirmatory factor analysis of adverse effects. (AE, adverse effects).

Then we examined the adverse effects of Smartphone use on the Learning Foreign Language Scale. CFA results showed unacceptable indices: χ2 (9) =102.46; χ2/df = 11.39; *p* < 0.001; CFI = 0.92; TLI = 0.86; RMSEA (90% confidence interval) =0.144[0.120, 0.170]; SRMR = 0.07. According to the MI values, we built correlations between the errors of two items. Besides, the factor loading of one item was below 0.45. Hence this item was also deleted. Then the CFA result indicated the following fit indices: χ2(4) =8.88; χ2/df = 2.22; *p* = 0.064 (*p* > 0.05), CFI = 0.99; TLI = 0.99; RMSEA (90% confidence interval) =0.049 [0.000, 0.094]; SRMR = 0.02. Then the revised adverse effects model was accepted, as shown in Figure 3. The factor loading of each item for adverse effects ranged from 0.53 to 0.86, all above 0.5. The AVE was 0.51, and the CR value was 0.83, signaling an excellent convergent validity. The total Cronbach alpha value of the scale was 0.84, showing good internal consistency.

### 4.3. Predictive effects of digital nativity and Smartphone usage

Table 3 shows that four factors of C-DNAS were all significantly related to the second-order factor of favorable Smartphone usage. But “grow up with technology” and “comfortable with multitasking” are not significantly correlated with adverse effects.

**Table 3:** The correlations among C-DNAS and SULFLS.

Factors	GC	RW	LS	FSU	AE
GUT	0.22**	−0.06	0.30**	0.19**	0.07
CM	0.19**	0.07	0.29**	0.23**	0.03
RGC	0.09*	0.11*	0.20**	0.19**	0.17**
TIGR	0.25**	0.04	0.31**	0.25**	0.26**

**Correlation is significant at the 0.01 level. *Correlation is significant at the 0.05 level. GUT, grow up with technology; CM, comfortable with multitasking; RGC, reliant on graphics for communication; TIGR, thrive on instant gratifications and rewards; GC, general contribution; RW, reading and writing; LS, listening and speaking; FSU, favorable Smartphone Usage; AE, adverse effects.

**Figure 4:**
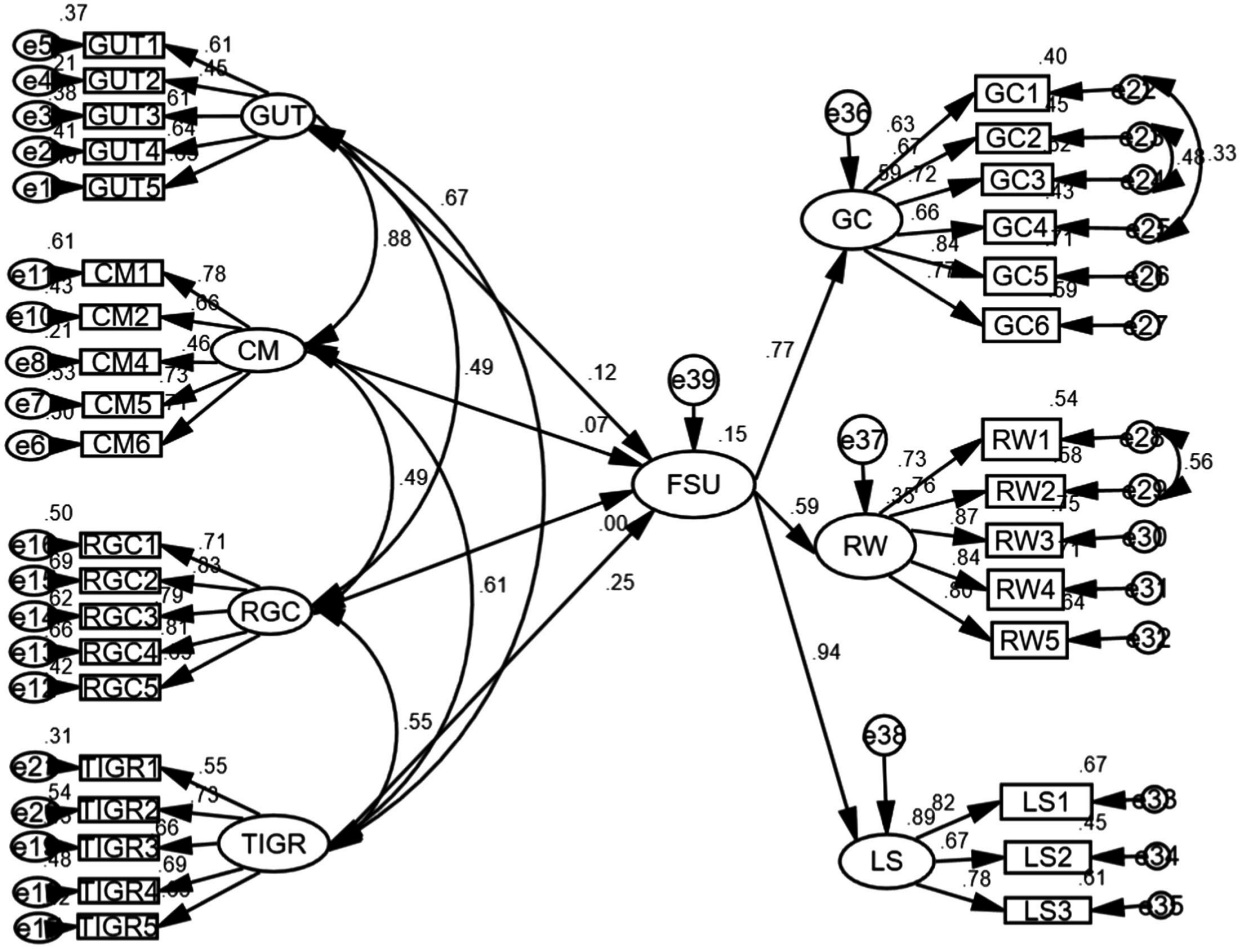
The SEM results of digital nativity and favorable Smartphone usage.

First, a structural equation modeling was conducted to examine how the four antecedents of digital nativity generated positive effects on Smartphone usage of English learning. The model fit indices were acceptable, which were: χ2 (511) = 1336.86; χ2/*df* = 2.62; *p* < 0.01; CFI = 0.90; TLI = 0.89; RMSEA (90% confidence interval) = 0.057 [0.053, 0.061]; SRMR = 0.071. As shown in Figure 4, “grow up with technology” and “thrive on instant gratifications and rewards” significantly predict favorable Smartphone usage positively, with path coefficients separately as 0.12 and 0.25.

**Figure 5:**
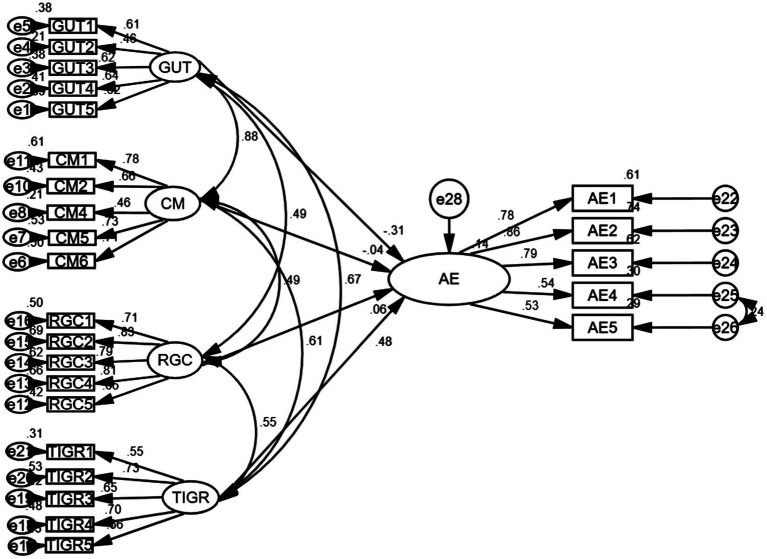
The path between digital nativity and adverse effects of Smartphone usage.

The structural equation modeling indices between digital nativity and adverse effects are: χ2 (264) = 698.57; χ2/df = 2.65; CFI = 0.91; TLI = 0.90; RMSEA (90% confidence interval) = 0.057[0.052, 0.063]; SRMR = 0.06; *p* < 0.00. In this model, as shown in Figure 5, “grow up with technology” negatively predicts the adverse effects to a significant level (β = −0.31, *p* < 0.01), and “thrive on instant gratifications and rewards” positively explains the variations of adverse effects (β = 0.48, *p* < 0.01). However, in both models, “comfortable with multitasking” and “reliant on graphics” fail to predict either the favorable Smartphone usage or the adverse effects, because none of the path coefficients is significant.

## 5. Discussion

The study aimed to examine the predictive effects of digital natives on Smartphone use in Chinese EFL contexts. Digital nativity was found to be a four-factor structure, including “grow up with technology,” “comfortable with multitasking,” “reliant on graphics for communication” and “thrive on instant gratifications and rewards,” in line with the results found in other learning contexts (Chen et al., 2016; Teo, 2016; Çoklar et al., 2017; Yurdakul, 2018; Wang et al., 2019; Calvo-Ferrer, 2020; Huang et al., 2021; Zhao and Zhao, 2021). These factors resonated Prensky (2001) summary for the typical features of digital natives and supported the contention that digital nativity is a psychometric property instead of characteristics defined by age discrepancy (Teo, 2013; Huang et al., 2021; Zhao and Zhao, 2021). As was noted by Teo (2013), this result shows the degree to which the participants perceive themselves as technological acquaintances, with higher scores designating more tendency toward digital nativity. The applicability of the scale as an effective instrument to examine an individual’s digital nativity was also tested.

One interesting result is the second-order construct of favorable Smartphone usage, with the three positive dimensions (“general contribution,” “reading and writing,” “listening and speaking”) loaded on a higher factor, named favorable Smartphone usage. Our findings revealed that the three advantageous factors converged on one inclusive construct, enclosing the active use of Smartphones as effective and beneficial in English learning. It coincides with Şad et al. (2020) research results, in which both positive and negative effects of Smartphone use were examined. However, students showed a less tendency to use Smartphones for their reading and writing skills, which might be caused by the small screen size (Şad et al., 2020) and inconvenience of typing on the delicate gadget. Therefore, the physical feature of Smartphones might be the reason for the participants to conduct less reading or writing activities. Another possible reason is the relative negligence of English writing in university education. Gao (2007) pointed out that although writing plays an integral role in the four basic language skills, it has long been neglected in Chinese universities. In addition, the adverse effects of five items formed a distinct factor, including the entertaining nature and distractive activities while using Smartphones (Metruk, 2022). Besides, the easy access to information with the help of Smartphone also plays a harmful effect on students’ deep cognition. The result is similar to the previous argument of Şad et al. (2020) that students use their Smartphones to improve their English skills generally. Still, the easy access to English language content *via* Smartphones generates negative effects on their deep thinking and cognition. Therefore, while recognizing the benefits of Smartphone usage to improve English learning, learners should also be cautious for the potential harms and detriments of Smartphones usage. Generally speaking, it is essential to explore both positive and negative impacts of Smartphone use for educational purposes (Baran, 2014).

Another significant contribution of this study is the examination of the comprehensive path impacts of four digital native attributes on the favorable use and the adverse effects. The path evaluation results showed a significant positive predicting effect from “grow up with technology” to favorable use of Smartphones in English learning. As Teo (2016) found, digital natives who grew up with the advancement of technology considered themselves skillful technologically, and those who possessed more characteristics of digital natives used technology longer. It is reasonable to posit that those who show more attributes of digital nativity tend to be more adept in technology usage since they have more technological experience with more substantial technology confidence. As previous researchers claimed (Frand, 2000; Oblinger and Oblinger, 2005), digital natives prone to possess better cognitive capacities. Hence, students who have access to technology frequently are more likely to adopt appropriate technological tools according to the nature of different learning tasks and value the usefulness of mobile technology. In other words, college students with more experience with technology access might also report that they use Smartphones to benefit English learning. Therefore, it is reasonable to argue that college students with sufficient technological experience benefit more from using Smartphones in their English learning processes.

Our study reported that “grow up with technology” negatively influenced the adverse effects. This might be attributed to the claim by Prensky (2001) that human brains may grow and change in response to environmental stimulation. The brain structures of digital natives might display neuroplasticity and malleability as an adaptation to the increasingly digitalized world (Prensky, 2001). As such, digital natives growing up with technological advancement command a repertoire of facilitating strategies (Tapscott,1999; Prensky, 2001) for their English learning with mobile assistance and develop anti-distraction (antijamming) mechanisms to minimize the adverse effects. For example, Wang et al. (2019) reported that “grow up with technology” have no significant predicting impact on Smartphone addiction. Their study revealed a negative path coefficient between this attribute of digital nativity and Smartphone addiction, although insignificantly. What’s more, a similar research result showed that none of the digital nativity attributes could be used as a predictor for students’ disengagement with the educational system (Calvo-Ferrer, 2020). Hence, the “grow up with technology” feature can be taken as a beneficial factor (Tapscott, 1999; Prensky, 2001) in alleviating language learners’ sufferings from the adverse effects of Smartphone usage. It is taken as an affirmative feature (Aharony and Gazit, 2019), positively contributing to the favorable use and negatively affecting the adverse effects.

However, “thrive on instant gratifications and rewards” displayed a much more mixed influence on Smartphone usage, which has a significant predicting effect on both favorable and adverse use of Smartphones. On the one hand, the convenience of Smartphones can readily satisfy learners’ instant needs and provide immediate feedback (Kukulska-Hulme and Viberg, 2018; Şad et al., 2020). Hence digital learners who thrive on instant gratifications have a strong tendency to adopt Smartphones as a valuable tool to English practice positively. This positive aspect is in line with Jiang and Zhang (2020) findings that English learners with proper guidance can improve communication efficiency and writing skills by using mobile devices. Consequently, mobile devices have become an ideal tool for fulfilling the needs of modern digital learners, which explains the positive path from “thrive on instant gratifications and rewards” to positive Smartphone usage. On the other hand, concerning the higher predicting role “thrive on instant gratifications and rewards” played in adverse use of Smartphones, a preference for quick responses by using mobile phones may lead up to this adverse effect. According to the research result of Chen et al. (2016), the preference for quick access to information may distract students from their academic engagement and interfere with students’ academic decisions. Therefore, the priority of efficient need satisfaction may incur the sacrifice of long-term mastery of English among today’s digital natives and lead to superficial achievements in the short-term (Calvo-Ferrer).

We also found the insignificant predicting effects between multitasking and Smartphone usage in either positive or negative ways. The result echoes relevant research findings, showing that multitasking does not significantly predict either Smartphone addiction (Wang et al., 2019), technological disengagement (Calvo-Ferrer, 2020), or leisure decisions in a school-leisure conflicting environment (Chen et al., 2016). However, this finding contradicts Burak (2012) result that students’ multitasking is significantly related to lower online learning outcomes. One possible reason might be that Smartphone usage for English learning does not involve multitasking processes. As noted by Teo (2013), the digital natives’ assessment did not distinguish between various digital environments, such as computers, video games, Smartphones, etc. Smartphones usually do not allow the operation of many tasks within a small gadget. The absence of multitasking operations may be the reason for insignificant predicting effects. However, the robustness of this research finding is still open for further tests. More empirical research is needed to test the influencing mechanism of multitasking on digital natives learning behaviors with high-tech devices.

Besides, the characteristics of “reliant on graphics for communication” did not predict students’ adaptive usage of Smartphones or maladaptive functions. This outcome coincides with the research results that the preference for graphics in communication cannot statistically predict either the short-term or the long-term vocabulary retention and disengagement of the education system in a game-based online environment (Calvo-Ferrer, 2020). But it contradicts the evidence in previous research, which found this feature of the digital nativity to be a significant antecedent for Smartphone addiction (Wang et al., 2019). This may be due to the nature of language learning. Listening, speaking, reading, and writing are usually considered core skills for language learning. However, none of the learning processes was directly associated with using pictures or graphics. Graphics may facilitate learners’ comprehension of abstract knowledge or the absorption of content information (Lowe and Pramono, 2006) instead of language structures. However, it is noted that this explanation warrants further empirical investigations.

## 6. Conclusion

This study examined the predictive effects of multi-dimensional factors of digital nativity on both favorable and adverse usage of Smartphones in Chinese college English learning. It was found that (1) digital nativity consisted of four factors; (2) a second-order factor of favorable Smartphone usage with first-order adverse effects in English learning was confirmed; (3) “Grow up with technology” significantly positively predicted favorable Smartphone use but negatively predicted adverse effects. “Thrive on instant gratification and rewards” significantly predicted both favorable and adverse effects in a positive way. However, “comfortable with multitasking” and “reliant on graphics for communication” could not predict Smartphones usage in English learning. Thus, the four digital attributes exerted different effects on Smartphones usage for Chinese college students’ English learning. Findings reveal the significance of defining digital nativity as an intricate and multifaceted construct (Wang et al., 2019). The different predictive effects of the four factors on Smartphone usage for both positive and negative purposes help us better understand how individuals’ psychological characteristics affect their behavior technologically advanced learning environment (Huang et al., 2021; Zhao and Zhao, 2021).

Our results provide some pedagogical implications for EFL learning and teaching in digital environments. First, teachers are suggested to be fully aware of the technological savviness of modern digital natives and proactively explore, select, and introduce high-quality digital resources (Kesharwani, 2020) for English learning to the students. Thus, realizing the role transfer from instructor and transmitter to the facilitator (Tapscott, 1999), information provider, communicator, knowledge-building supporter, and manager (Gonzalez, 2010) is essential and helpful. Teachers should give more trust to modern learners in exploring the beneficial functions of mobile devices and avoiding harsh effects. Thus, teachers are suggested to create a more encouraging environment and readily take advice from resourceful students to nurture friendly and generative surroundings. Second, the online group discussion forum is encouraged to be established so that answers to language learning questions are elicited from teachers and peer students, thus forming a collaborative learning environment (Oblinger and Oblinger, 2005; Tapscott, 2009). A student-centered (Jones and Shao, 2011) collaborating learning platform can relieve teachers’ heavy burdens of answering too many questions and impel students’ negotiation and concertation in dealing with language learning problems through peer learning. Finally, sufficient instructions in avoiding the adverse effects of technology (Kirschner and De Bruyckere, 2017) are required as an indispensable part of mobile-assisted language learning processes.

There are still some limitations in our study despite the informative results. First, this study is cross-sectional, which allows us to draw tentative conclusions on the causal effects of digital nativity on Smartphone usage. It is suggested to apply longitudinal designs to test structural relationships and their stability across a period. Second, this study used self-report questionnaires, which might limit the objectivity of the results since respondents might need to answer the questionnaires accurately. In response to this issue, other approaches to data elicitation (e.g., interview, reflective journal, and immediate stimulative recall after a task) are encouraged to collect multiple sources for triangulating the results. Finally, the English proficiency levels of participants and more individual variables need to be considered when exploring relationships between digital natives and their Smartphone usages, such as digital literacy, self-efficacy, learning motivation, self-regulation, and learning contexts.

## Data availability statement

The raw data supporting the conclusions of this article will be made available by the authors, without undue reservation.

## Ethics statement

The studies involving human participants were reviewed and approved by Ethical Committee of the Northeastern University. The patients/participants provided their written informed consent to participate in this study.

## Author contributions

LT and LH worked on research design conception, data analysis, and manuscript draft. FG worked on data collection and analysis. All authors contributed to the article and approved the submitted version.

## Funding

This research was funded by the Social Science Funds of Liaoning Province in China grant number L21AYY005.

## Conflict of interest

The authors declare that the research was conducted in the absence of any commercial or financial relationships that could be construed as a potential conflict of interest.

## Publisher’s note

All claims expressed in this article are solely those of the authors and do not necessarily represent those of their affiliated organizations, or those of the publisher, the editors and the reviewers. Any product that may be evaluated in this article, or claim that may be made by its manufacturer, is not guaranteed or endorsed by the publisher.
